# Prey Density Threshold and Tidal Influence on Reef Manta Ray Foraging at an Aggregation Site on the Great Barrier Reef

**DOI:** 10.1371/journal.pone.0153393

**Published:** 2016-05-04

**Authors:** Asia O. Armstrong, Amelia J. Armstrong, Fabrice R. A. Jaine, Lydie I. E. Couturier, Kym Fiora, Julian Uribe-Palomino, Scarla J. Weeks, Kathy A. Townsend, Mike B. Bennett, Anthony J. Richardson

**Affiliations:** 1 Project Manta, The University of Queensland, St Lucia, QLD, 4072, Australia; 2 School of Biomedical Sciences, The University of Queensland, St Lucia, QLD, 4072, Australia; 3 Biophysical Oceanography Group, School of Geography, Planning and Environmental Management, The University of Queensland, St Lucia, QLD, 4072, Australia; 4 Manta Ray and Whale Shark Research Centre, Marine Megafauna Foundation, Tofo Beach, Inhambane, Mozambique; 5 Aix-Marseille Université, Mediterranean Institute of Oceanography (M.I.O), UMR 7294, UR 235, Campus de Luminy, Case 901, 13288, Marseille, Cedex 09, France; 6 Lady Elliot Island Dive Shop, Lady Elliot Island Eco Resort, Great Barrier Reef, QLD, Australia; 7 CSIRO Oceans and Atmosphere Flagship, EcoScience Precinct, Brisbane, QLD, 4102, Australia; 8 Centre for Applications in Natural Resource Mathematics, School of Mathematics and Physics, The University of Queensland, St Lucia, QLD, 4072, Australia; National Taiwan Ocean University, TAIWAN

## Abstract

Large tropical and sub-tropical marine animals must meet their energetic requirements in a largely oligotrophic environment. Many planktivorous elasmobranchs, whose thermal ecologies prevent foraging in nutrient-rich polar waters, aggregate seasonally at predictable locations throughout tropical oceans where they are observed feeding. Here we investigate the foraging and oceanographic environment around Lady Elliot Island, a known aggregation site for reef manta rays *Manta alfredi* in the southern Great Barrier Reef. The foraging behaviour of reef manta rays was analysed in relation to zooplankton populations and local oceanography, and compared to long-term sighting records of reef manta rays from the dive operator on the island. Reef manta rays fed at Lady Elliot Island when zooplankton biomass and abundance were significantly higher than other times. The critical prey density threshold that triggered feeding was 11.2 mg m^-3^ while zooplankton size had no significant effect on feeding. The community composition and size structure of the zooplankton was similar when reef manta rays were feeding or not, with only the density of zooplankton changing. Higher zooplankton biomass was observed prior to low tide, and long-term (~5 years) sighting data confirmed that more reef manta rays are also observed feeding during this tidal phase than other times. This is the first study to examine prey availability at an aggregation site for reef manta rays and it indicates that they feed in locations and at times of higher zooplankton biomass.

## Introduction

Large tropical and sub-tropical marine animals use various strategies to meet their energetic requirements from a relatively nutrient-poor environment. Leatherback turtles *Dermochelys coriacea* aggregate at known “hotspots” of prey availability [1], Abbott’s boobys *Papasula abbotti* alter their foraging behaviour via prey switching [2], and humpback whales *Megaptera novaeangliae* undertake vast annual migrations (>10,000 km) to exploit nutrient-rich polar waters [3]. Planktivorous elasmobranchs such as whale sharks *Rhincodon typus* and manta rays *Manta birostris* and *M*. *alfredi*, whose thermal thresholds prevent them from foraging in food-rich polar waters [4], must spend the majority of their life in oligotrophic tropical and sub-tropical waters, where they are also observed feeding [5–8].

Biological, chemical and physical factors operating over a variety of spatio-temporal scales impact the distribution and density of zooplankton [9–11]. Tidal currents, coupled with bathymetry, concentrate plankton and provide important feeding areas for marine animals [6]. Whale sharks in Ningaloo Reef [12] and basking sharks off headlands in southern England [13] feed where tidal flows create dense concentrations of zooplankton. Additionally, research in Komodo National Park in Indonesia [6] and both the southern and northern Great Barrier Reef (GBR) indicates that currents and tides influence the presence and behaviour of reef manta rays [5, 14], but there has been no study of the feeding and prey availability at any aggregation site for reef manta rays.

Traditional methods for examining a species diet, such as stomach content analysis, are not appropriate for large threatened marine fishes [15]. Thus non-lethal techniques including direct observation [16] and biochemical analyses are preferred for inferring dietary preference and trophic position [17]. Manta rays are commonly observed in surface waters using “ramjet feeding” to feed on zooplankton [5, 6]. The animals swim with their mouths open and use their cephalic lobes to direct zooplankton into the wide opening, where it is filtered using specialised gill plates [18]. By distinguishing feeding and non-feeding behaviour, and measuring the zooplankton present during these events, the relationship between feeding dynamics and prey availability and density can be elucidated [13]. Dense prey concentrations are a critical factor in feeding for planktivores, as the energetic cost of feeding needs to be balanced by a higher energy intake. For some species, prey availability needs to reach a minimum density to trigger feeding. Prey density thresholds have been estimated for fin whales *Balaenoptera physalus* [19], right whales [20], whale sharks [7] and basking sharks [16], but are unknown for manta ray species.

Prey size and composition can be important in predicting planktivore feeding. Seasonal aggregations of whale sharks coincide with swarms of neritic euphausiids (krill) at Ningaloo Reef in Western Australia [12], seasonal fish spawn off Gladden Spit in Belize [21], and summer increases in zooplankton in the Sea of Cortez in Mexico [7]. Foraging basking sharks off southern England feed on patches with copepods 50% larger than other areas [22] and stomach content analysis of whale sharks revealed consumption of large-bodied sergestids and mysids [23]. Aggregations of reef manta rays, such as those in the Maldives [8], Western Australia [24], eastern Australia [5] and Indonesia [6], are all related to enhanced productivity, however little is known about the zooplankton density, size or composition when reef manta rays are feeding.

Lady Elliot Island (LEI) in the southern GBR is the largest known aggregation site for reef manta rays off eastern Australia [25]. Jaine et al. [5] examined the abundance and behaviour of reef manta rays in relation to environmental factors using daily sighting records logged by the dive operator at LEI. Their results indicated that tidal phase was one of the important predictors of foraging behaviour. However, questions remained as to the food environment and fine-scale oceanographic drivers in the area. The current study investigated the zooplankton community and associated oceanographic dynamics that might influence foraging behaviour of reef manta rays around LEI. We aimed to describe the biomass, taxonomic composition and size structure of the zooplankton community in relation to reef manta ray behaviour; to establish whether reef manta rays exhibit critical feeding thresholds in relation to prey density; to investigate potential oceanographic drivers of zooplankton dynamics; and to test whether the oceanographic drivers identified in zooplankton analysis were concordant with patterns from long-term sighting records provided by the local dive operator.

## Methods

**Fig 1:**
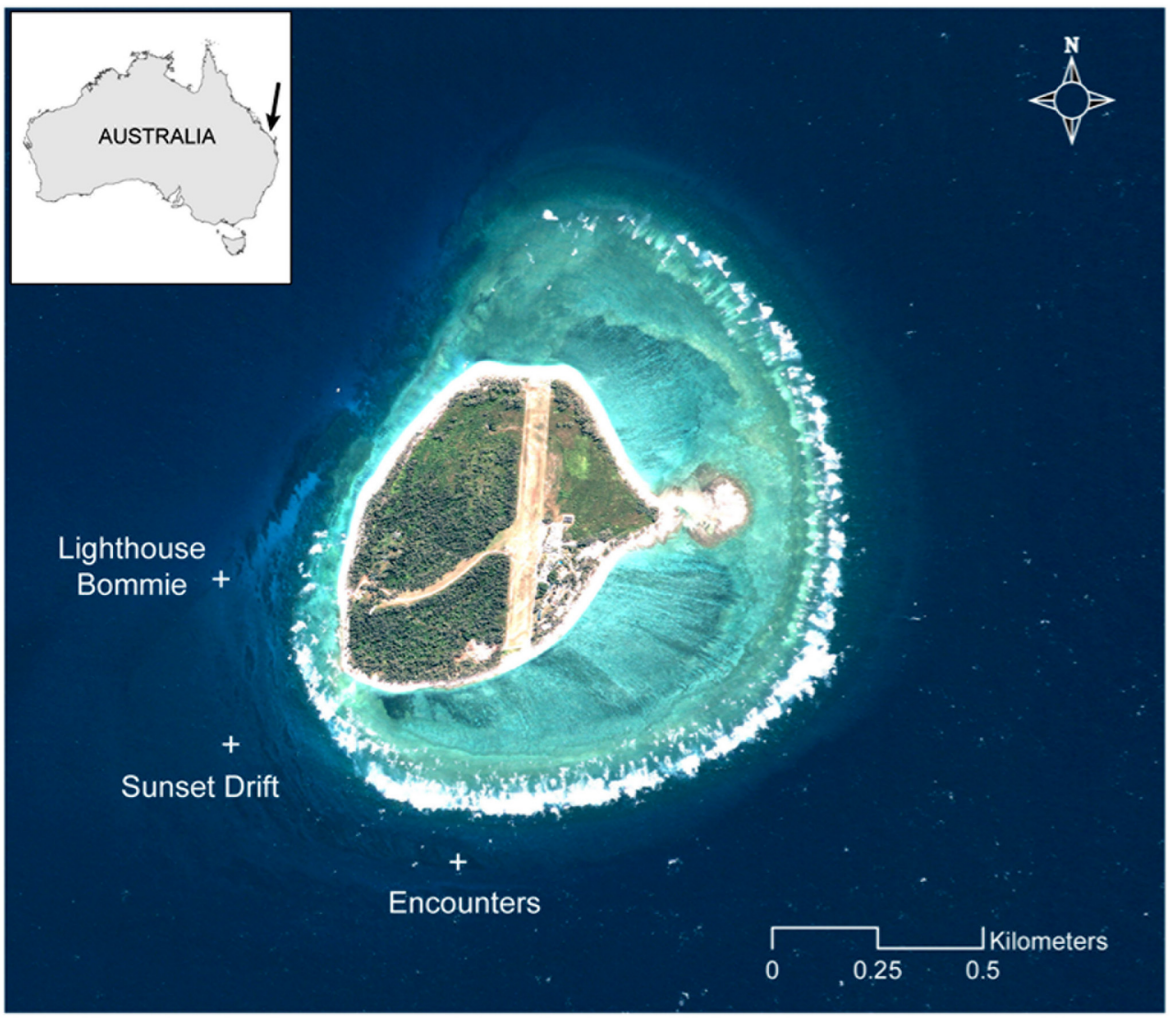
Sampling sites of Lady Elliot Island. Map of Lady Elliot Island (24°07’S, 152°43’E) in the southern Great Barrier Reef, Australia. Plus signs indicate the three *in-situ* sampling sites of Lighthouse Bommie, Sunset Drift and Encounters. High-resolution image obtained from the Quickbird satellite (Geoimage Pty Ltd., www.geoimage.com.au) and processed using ArcGIS 10.

Lady Elliot Island (24°07’S, 152°43’E) is a coral cay located in the southern GBR on Australia’s east coast (Fig 1). Sampling focused on the south-west region of LEI where 85% of foraging behaviour is observed [5]. Three sites were sampled every ~90 min, from 7 am to 5 pm, during the austral summer (trip 1: 10–13 February) and winter (trip 2: 12–20 June 2014, Fig 1). The work was conducted under GBRMPA permit G12/35136.1. Properties of the water column were measured using a SBE 19plus V2 Conductivity Temperature Depth and Fluorescence (CTD-F) SeaCAT profiler (Sea-Bird Electronics, USA). Near-surface temperature, salinity and fluorescence from the CTD-F were taken as the mean values of the profile from 1–3 m. A 200 μm mesh zooplankton net was towed for 2.5 minutes against the prevailing current, at a speed of ~2 knots. The net was towed within 1–2 m of the sea surface, the depth at which reef manta rays are commonly observed feeding at LEI. The net had a 50 cm diameter mouth opening fitted with a digital flowmeter to calculate the volume of water filtered during each tow. Flowmeter calibration was performed prior to each field trip. During the tow, observations of reef manta ray abundance and behaviour (feeding, non-feeding, absent) were recorded. Zooplankton samples were preserved using buffered formalin to achieve a 5% concentration.

### Zooplankton biomass, taxonomy and size

Zooplankton samples (n = 90) were split using a Folsom splitter [26]. One half was oven-dried at 70°C for 24 hrs to obtain the dry weight as a measure of biomass. Zooplankton biomass (mg m^-3^) was calculated by dividing the dry weight (mg) by the volume of water filtered (m^3^).

**Fig 2:**
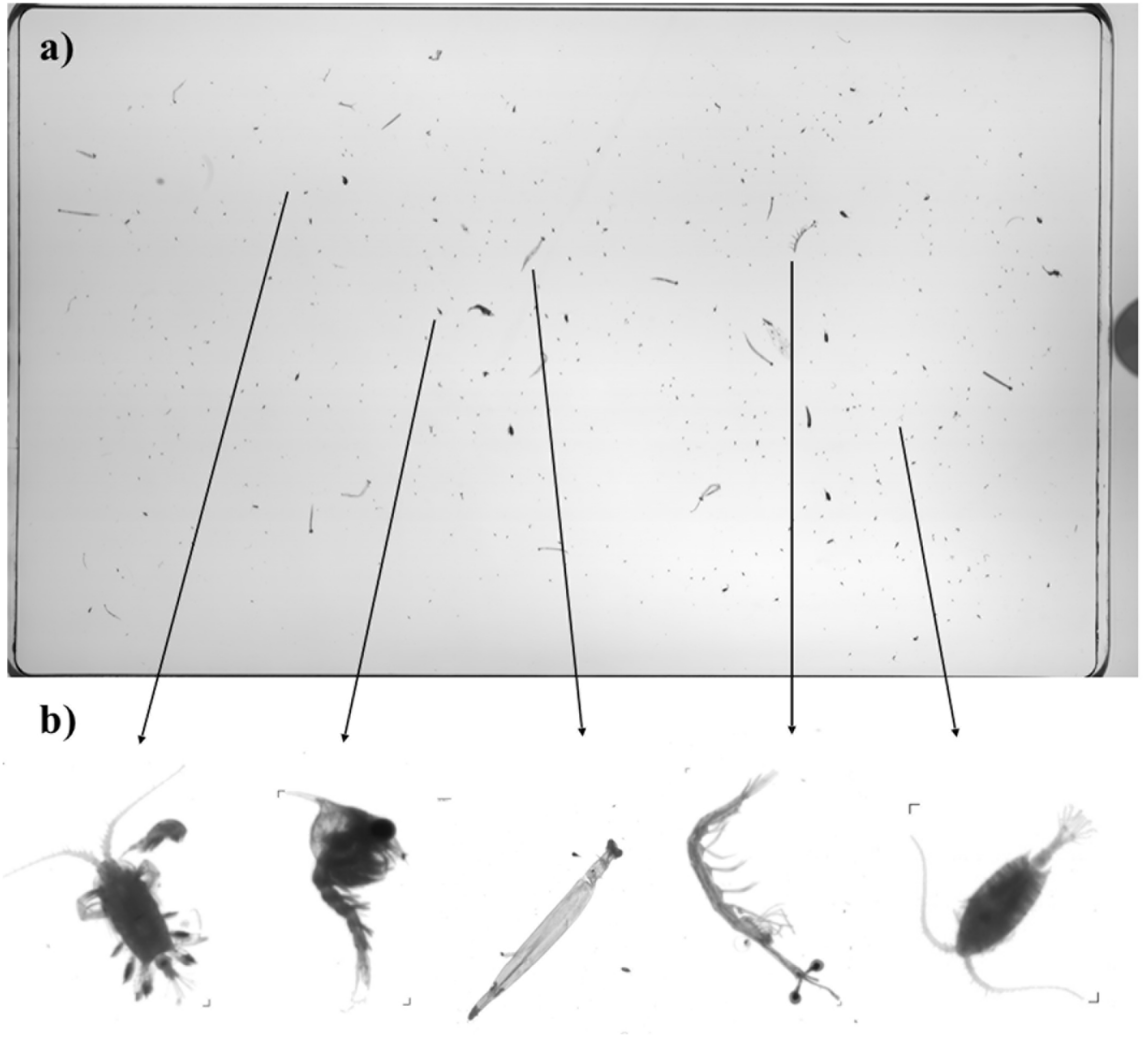
ZooScan system analysis and output. Using ZooScan to measure the size and composition of zooplankton at Lady Elliot Island: a) Scanned image of a zooplankton subsample, and b) examples of digitally-separated vignettes.

We used the second half of the sample to investigate the size and composition of individuals in the zooplankton community using a Hydroptic v3, EPSON Perfection V700 Flatbed) 2400 dpi ZooScan system (n = 50). The ZooScan system is a high resolution, waterproof scanner that digitises particles for size and biovolume measurements [27]. This approach also uses an artificial neural network identifier to identify automatically the broad types of zooplankton present. A 1% subsample of each sample was prepared using a Stempel pipette and placed on the scanning tray. Particles were manually separated to avoid overlap. The sample was then scanned and particles were extracted into vignettes, and then categorised into broad taxonomic groups (24 groups) using Plankton ID software (Version 1.2.6) and manual validation (Fig 2). Objects identified as sand, fibre, detritus, bubbles or shadows were excluded from further analysis. For visualisation, taxa that comprised <2% of the total abundance were grouped as “Other”.

To analyse the size structure of the zooplankton community, a size distribution of the particles in the water column, known as the Normalised Biomass Size Spectra (NBSS), was produced [27]. Size measurements obtained from ZooScan for each particle were converted to a spherical biovolume (SBv). Each particle was then assigned to one of 50 logarithmic size (biovolume) categories based on its SBv. The sum of the SBv of the particles in each size class (mm^3^) was then standardised by the fraction of sample scanned and the volume of water filtered (m^3^), and normalised by dividing this value by the width of the size (biovolume) class (mm^3^). Both axes of the NBSS use a logarithmic scale [27].

All size spectrum data have been lodged with the Australian Zooplankton Database, curated by CSIRO [28]. Following a two-year moratorium, these data will be openly accessible to the research community through the Australian Ocean Data Network (AODN).

### Theoretical estimate of prey density threshold

To calculate the threshold prey density for reef manta rays and compare with our in situ estimate, we adapted an approach used for basking sharks by Sims [16]. This method provides a first-order approximation of an animal’s energy requirements and the prey density required to trigger feeding. It makes a number of assumptions. An average-sized reef manta ray was assumed to have a disc width of ~3.5 m (~3 m for males, ~4 m for females), a mouth opening of 0.3 m^2^, and a weight of ~100 kg based on morphometrics from southern Africa [29]. From personal observations, we assumed a swimming speed of 2 knots (3704 m hr^-1^) when feeding. Calculations for routine metabolic rate (422.98 kJ hr^-1^) were based on the metabolic-weight relationship for sharks provided by Parsons [30]:

$$MR\;=\;(\text{68}.9+177.8\;\times \;W)\;\times \;\frac{\text{3}.25}{W\;\times \text{ 24}}\;$$

Where *MR* is metabolic rate in calories kg^-1^ day^-1^ and *W* is weight in kg. In the absence of specific information for reef manta rays, we applied an efficiency rate of 80% to several calculations, as has been used for basking sharks, for: 1) buccal flow velocity from forward swimming speed [31]; 2) zooplankton filtration [32]; and 3) energy absorption [33]. The filtration rate of seawater by reef manta rays (888.96 m^3^ hr^-1^) was based on the swimming speed, mouth opening and filtration efficiency estimates. The routine metabolism was divided by the filtration rate to calculate the amount of energy required (0.744 kJ m^-3^) from the zooplankton to balance energy expenditure, accounting for the efficiency of energy absorption. The zooplankton community was assumed to comprise mostly copepods and other crustaceans, as has been observed in the current study, so the mean energy content was taken as that of copepods (5.04 kJ g^-1^ wet weight; adjusted to dry weight using a 0.171 conversion for crustaceans; [34]). The energy requirement was then divided by the energy content of the zooplankton to give the prey density threshold (mg m^-3^) required to balance energy lost. This method is based on the routine metabolic rate and does not account for extra energy requirements for foraging activity in reef manta rays, so it may underestimate the food density requirements of these animals.

### Lady Elliot Island sighting records

Daily reef manta ray sighting records logged by the LEI dive operator include data on the date, time and location of sightings, as well as associated environmental information including temperature, tides, wind direction and strength, surface conditions, current direction and strength, and water visibility. These data (n = 3126) were used as a long-term (May 2008 to September 2013) test of whether reef manta ray behaviour was influenced by the same environmental variables to those observed in our short-term field campaign.

### Statistical analysis

#### Zooplankton community and reef manta ray behaviour

A one-way analysis of variance (ANOVA) was used to test whether there were differences in biomass and abundance in relation to manta ray behaviour. Post-hoc analysis using Tukey pair-wise comparisons were then conducted to determine differences in biomass/abundance amongst behaviours.

Non-metric multidimensional scaling (MDS) was used to determine how different the zooplankton communities were according to manta ray behaviour (feeding or non-feeding). We conducted two separate analyses of the zooplankton data, as data transformation reduces the importance of abundant species [35]; the first was on the raw abundance data which is heavily influenced by the most abundant taxa, and the second was on presence-absence data and gives all species equal weighting. An analysis of similarity (ANOSIM) was used to test for differences in community composition between manta ray behaviours.

#### Critical foraging density threshold

A generalised linear model (GLM) with a binomial error structure was used to analyse manta ray behavioural response (non-feeding = 0, feeding = 1) to zooplankton biomass and/or biovolume as predictors. This is equivalent to a logistic regression. Non-significant variables were removed from the model using Akaike’s information criterion (AIC) values. The critical density threshold was taken as the zooplankton biomass at which the proportion of feeding was 0.5.

#### Oceanographic influences on zooplankton dynamics

An initial investigation of the relationship between zooplankton biomass at LEI and environmental predictors identified non-linear relationships. We thus used a Generalised Additive Model (GAM) approach, which uses scatterplot smoother to describe non-linearities [36]. We used the package mgcv in R, with smoothing parameters selected using the Restricted Maximum Likelihood (REML) method [37]. Variables were chosen to provide temporal (*Trip*) and environmental (*Tide*, *Temperature*, *Salinity* and *Fluorescence*) predictors of changes in zooplankton biomass. For the final model, non-significant variables were removed in a stepwise approach based on the lowest AIC values.

To analyse whether there was an impact of environmental variables on zooplankton community composition, a BIOENV was used, in the R package “vegan” [38]. BIOENV models the community distance matrix as a function of environmental variables (here *Tide*, *Trip*, *Temperature* and *Salinity*).

#### Long-term reef manta ray sighting records

We used the long-term sighting records of reef manta ray behaviours from LEI to test the relationships identified between zooplankton biomass and environmental predictors. Unfortunately the long-term sighting records do not include zooplankton biomass, but they do include whether reef manta rays were feeding or not. Our logic was that environmental predictors that lead to high zooplankton biomass during our two short (several weeks) field trips could manifest as a greater degree of feeding behaviour over the longer (~5 years) sighting records. All manta ray behaviours in the sighting records were categorised as either feeding or non-feeding and this was used in a GAM as a binomial response (non-feeding = 0, feeding = 1). Predictors selected were those that were found to be significant from the analysis of zooplankton and environmental predictors. For the final model, non-significant predictors were removed in a stepwise approach based on the lowest AIC values. Because of the nature of the response in logistic regression, r^2^ values are always lower than for typical linear regression [39]. There is also no agreement on how to calculate the r^2^ value in logistic regression—we have used the method of Tjur [40], which is the difference in the mean predicted probabilities of the two categories of the dependent variable.

All statistical analyses were carried out using the software package R (version 3.2.2, www.r-project.org). Assumptions of homogeneity of variance and normality for the analyses were assessed visually.

## Results

### Zooplankton dynamics and manta ray behaviour

**Fig 3:**
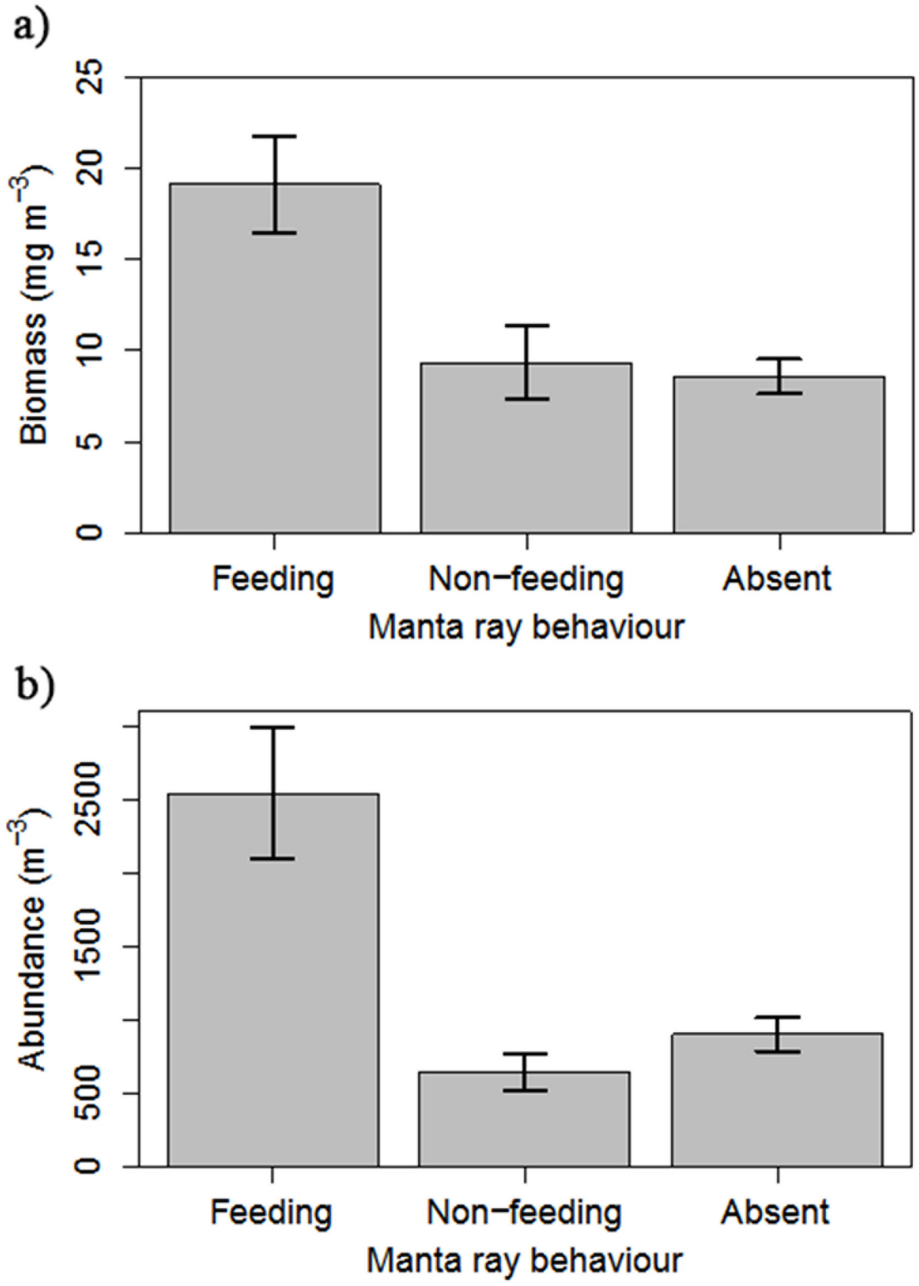
Zooplankton biomass and manta ray foraging behaviour. Zooplankton density in relation to reef manta ray behaviour at Lady Elliot Island: a) mean zooplankton biomass (mg m^-3^, ±standard error; feeding n = 17, non-feeding n = 12, absent n = 61); and b) mean zooplankton abundance (m^-3^ ±standard error; feeding n = 17, non-feeding n = 12, absent n = 21).

The biomass of zooplankton was significantly larger during reef manta ray feeding events (19.12 mg m^-3^, n = 17) than when reef manta rays were not feeding (9.33 mg m^-3^; TukeyHSD diff = -9.78, p<0.05) or were absent (8.59 mg m^-3^; TukeyHSD diff = 10.53, p<0.05; ANOVA F_(2,87)_ = 11.41, p<0.05) (Fig 3a). There was no significant difference in zooplankton biomass when reef manta rays were not feeding or were absent (TukeyHSD diff = 0.75, p = 0.95).

Abundance of zooplankton was also significantly higher during feeding events (2547 m^-3^) than when reef manta rays were not feeding (648 m^-3^; TukeyHSD diff = -1898.75, p<0.05) or were absent (906 m^-3^; TukeyHSD diff = -1641.66, p<0.05; ANOVA F_2,47_ = 11.48, p<0.05; Fig 3b). There was no significant difference in zooplankton abundance when reef manta rays were not feeding or were absent (TukeyHSD diff = -257.09, p = 0.84).

**Fig 4:**
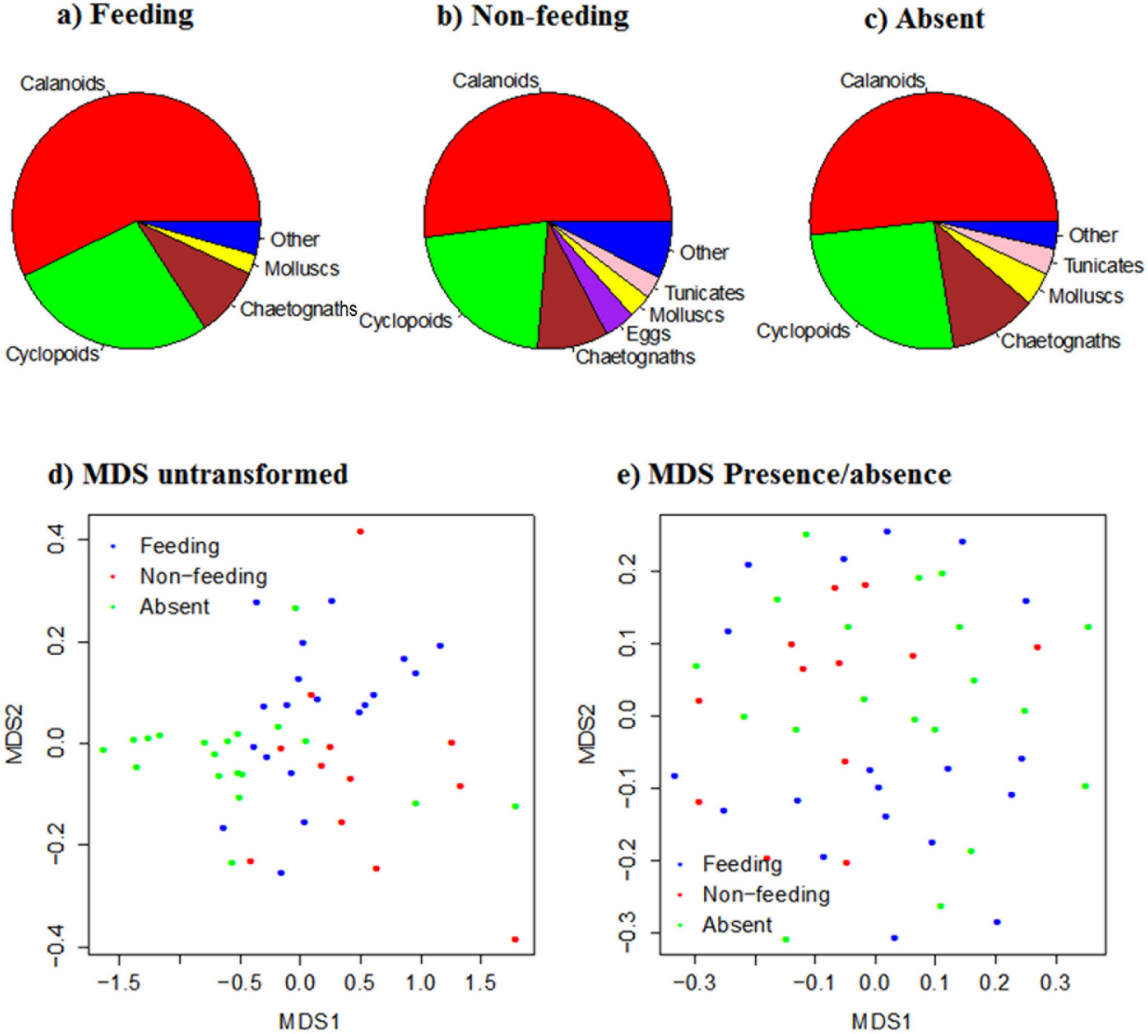
Zooplankton composition and manta ray foraging behaviour. Zooplankton composition at Lady Elliot Island in relation to reef manta ray behaviour showing little differences in composition: a) Feeding (n = 17), b) Non-feeding (n = 12), and c) Absent (n = 21). Nonmetric multidimensional scaling analysis of the community: d) with no transformation, and e) after a presence-absence transformation (n = 50).

Calanoid and cyclopoid copepods were the dominant zooplankton taxonomic groups, constituting ~75% of the community by number, irrespective of reef manta ray presence or behaviour (Fig 4). Other important taxa commonly found during both behaviours were chaetognaths and molluscs (Fig 4a, 4b and 4c). With raw abundance data, the MDS showed a significant difference in the zooplankton community among reef manta ray behaviours (ANOSIM; R = 0.24, p<0.05; Fig 4d). However, a presence-absence transformation of the data showed no significant difference in the zooplankton community among behaviours (ANOSIM; R = 0.04, p = 0.14; Fig 4e).

**Fig 5:**
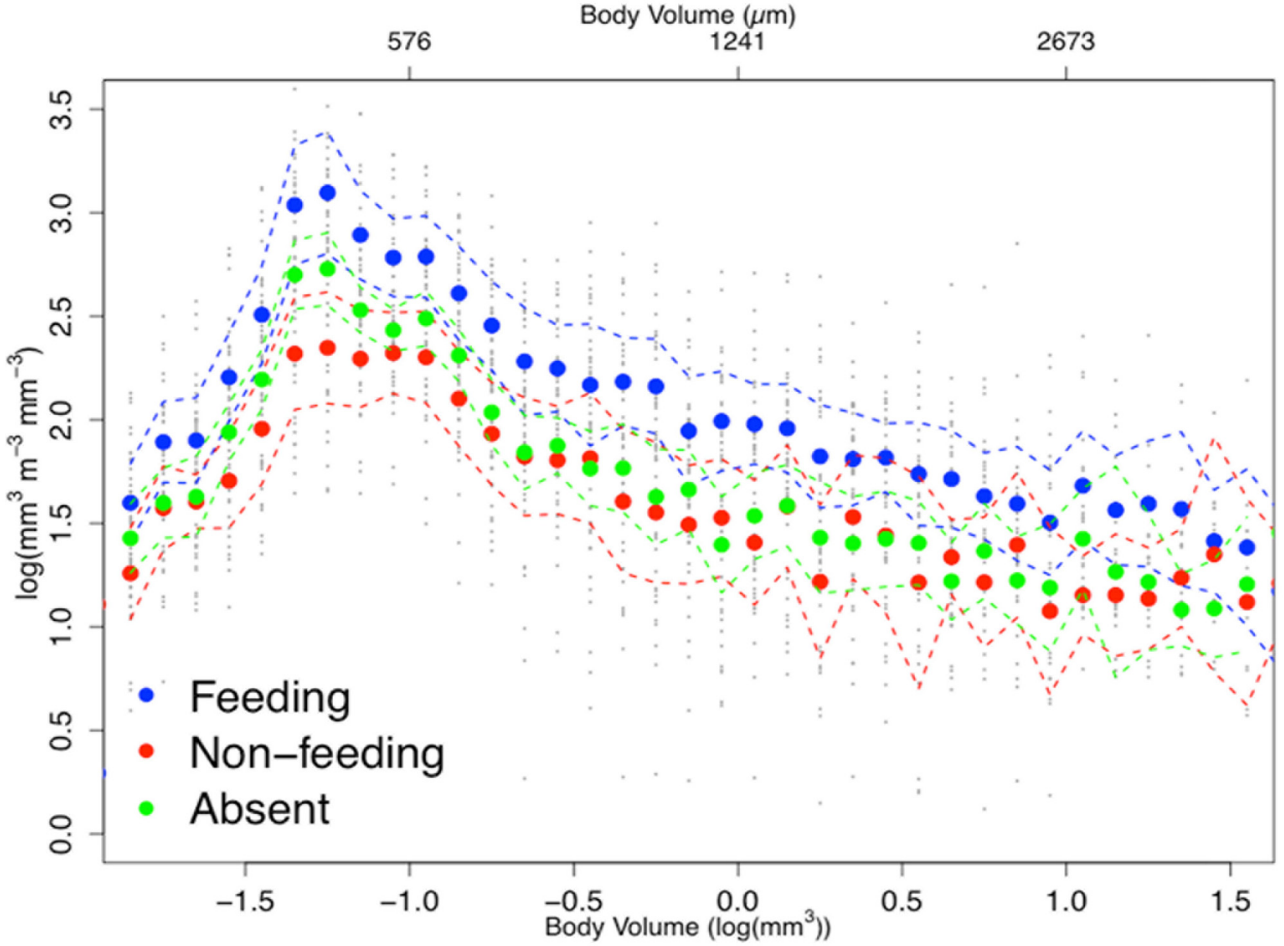
Zooplankton size spectra analysis. Normalised Biovolume Size Spectra of the zooplankton community showing higher biovolume across all size classes (body volume intervals) when reef manta rays are feeding (n = 17) than when non-feeding (n = 12) or absent (n = 21). Dashed lines represent 95% confidence intervals.

Analysis of the size structure of zooplankton from LEI revealed that the biovolume of zooplankton increased in all size categories when reef manta rays were feeding (Fig 5). There appeared to be significantly (non-overlapping confidence intervals) more zooplankton (larger total volume) across particle size categories during reef manta ray feeding events (mean = 1.89, se±0.09) than when they were not feeding (mean = 1.50, se±0.07) or absent (mean = 1.65, se±0.07). Small organisms dominated all samples, with a peak in total body volume of <500 μm in size, regardless of reef manta ray behaviour.

### Critical prey density threshold for reef manta rays

**Fig 6:**
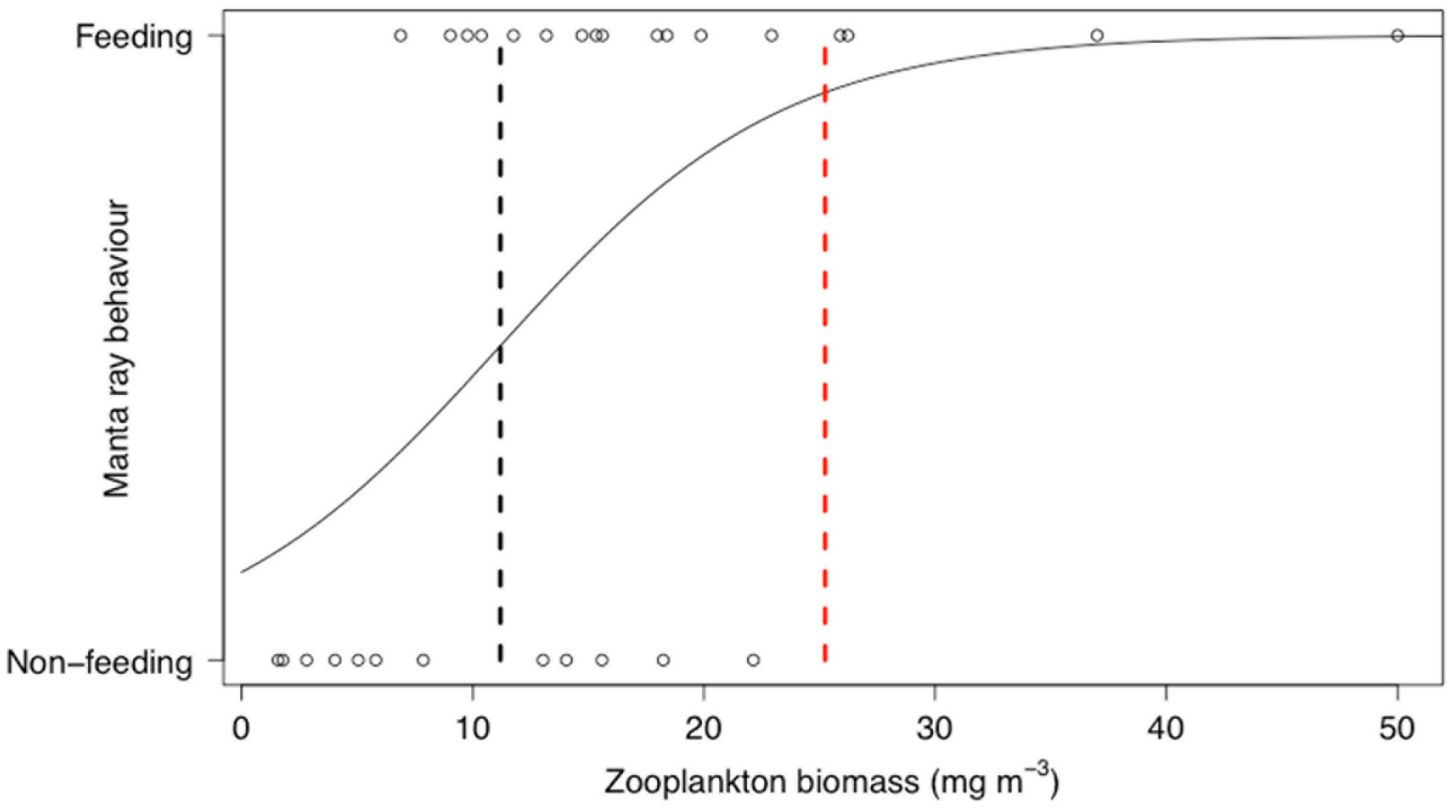
Critical prey density foraging threshold. Logistic regression of reef manta ray behaviour (feeding n = 17, non-feeding n = 12) in relation to zooplankton biomass (mg m^-3^). The black dashed line represents the critical density threshold of zooplankton biomass required to trigger reef manta ray feeding from *in-situ* sampling (11.2 mg m^-3^) and the red dashed line represents the theoretical calculation of the density threshold (25.24 mg m^-3^).

The logistic regression of reef manta ray behaviour against zooplankton biomass revealed that reef manta rays are significantly more likely to be feeding when zooplankton biomass is higher (Z_27_ = 2.28, p<0.05), but there was no significant effect of the size (biovolume) of zooplankton (Z_27_ = 0.33, p = 0.75). The critical prey density threshold for reef manta ray feeding was 11.2 mg m^-3^ (Fig 6). The theoretical density threshold calculated for reef manta ray feeding behaviour was estimated as 25.24 mg m^-3^, substantially higher than the 11.2 mg m^-3^ observed in the field (Fig 6).

### Potential oceanographic drivers

**Fig 7:**
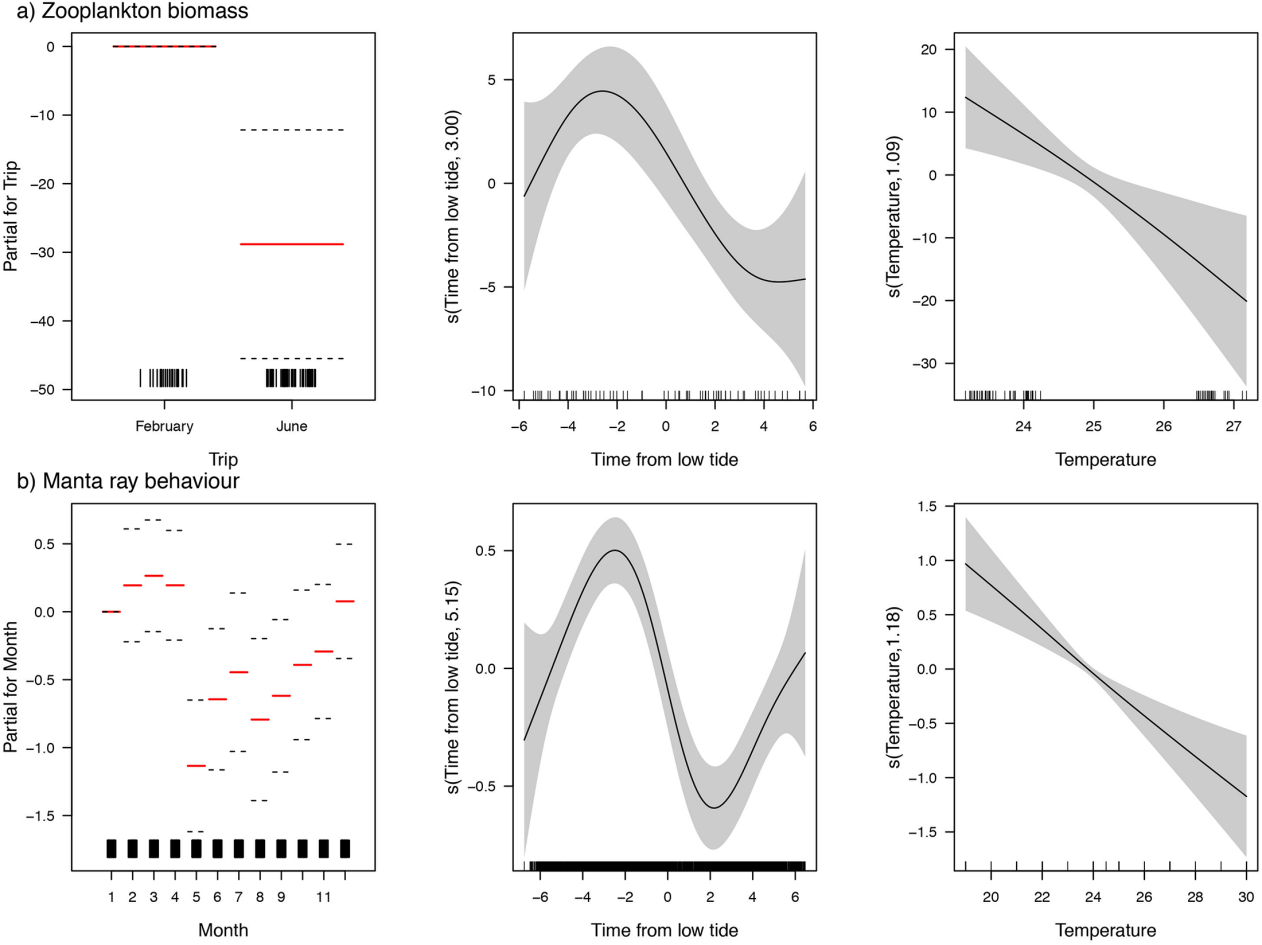
Oceanographic drivers of zooplankton biomass and manta ray feeding. Final generalized additive models for: a) Zooplankton biomass as the response and *Trip*, *Time to low tide* and *Temperature* as predictors (r^2^ = 42.8%, n = 70), and b) Manta ray behaviour (0 = non-feeding; 1 = feeding) as the response and *Month*, *Time to low tide* and *Temperature* as predictors (r^2^ = 5.3%, n = 2973). For each plot, the y-axis is a relative scale, and its magnitude reflects the importance of each variable. Dashed lines and error bars represent 95% confidence intervals.

The GAM of zooplankton biomass and environmental predictors revealed that *Trip*, *Time from low tide*, and *Temperature* were significant predictors of biomass at LEI (Fig 7a). Zooplankton biomass was significantly higher in the February than the June trip (t = 3.46, p<0.001). Zooplankton biomass was non-linearly related to *Time from low tide* (its estimated degrees of freedom is 3.00), with biomass peaking just over 2 hours before low tide, and declining before and after this time (F = 2.70, p<0.0001). Zooplankton biomass is approximately linearly related to *Temperature* (estimated degrees of freedom = 1.09), with significantly higher biomass found at cooler temperatures (F = 1.09, p<0.01). Zooplankton biomass was not significantly related to *Salinity* or *Fluorescence* (p>0.05).

The GAM based on the long-term sighting data with *Behaviour* as the response, confirmed that *Month* (akin to *Trip*), *Time since low tide*, and *Temperature* are all significant predictors. Further, the form of the relationships for the proportion of individuals feeding, an index of zooplankton biomass, is very similar to those for zooplankton biomass. From the long-term sighting records, the *Month* effect shows a lower proportion of individuals feeding in winter (June-August) (Fig 7b), as would be expected given we found lower zooplankton biomass in June *Trip* (Fig 7a). Further, based on the long-term sighting records, there is a greater proportion of reef manta rays feeding a couple of hours before low tide, consistent with the pattern we found for zooplankton biomass in our short-term field campaign. Finally, based on the long-term sighting records, there is a greater proportion of mantas feeding in cooler waters, consistent with the pattern we found for zooplankton biomass from our short-term field campaign.

Finally, the BIOENV analysis of the MDS on presence-absence data revealed that zooplankton community composition was not significantly related to *Tide* (R^2^ = 0.14, p = 0.21), *Trip* (R^2^ = 0.06, p = 0.39), *Temperature* (R^2^ = 0.4, p = 0.53) or *Salinity* (R^2^ = 0.005, p = 0.94). So the zooplankton community had a similar composition under different environmental conditions.

## Discussion

This is the first study to describe and quantify the *in-situ* prey availability at an aggregation site for reef manta rays. The species feeds at LEI when the biomass and abundance of zooplankton is significantly higher than at other times. There is no observable difference in the zooplankton community composition at LEI during feeding events, with all zooplankton taxa being more abundant. Changes in zooplankton biomass during this study were related to season, tide, and temperature. However, the composition of zooplankton did not change in response to these predictors, which suggests that the accumulation of high zooplankton biomass was driven by local oceanographic conditions rather than as a consequence of a different water mass bringing higher biomass of different zooplankton into the region.

The zooplankton prey density threshold for reef manta rays feeding at LEI was 11.2 mg m^-3^, with zooplankton biomass consistently higher when reef manta rays are feeding rather than not feeding. *In-situ* prey density thresholds have previously been calculated for a number of large planktivores including whales sharks, basking sharks, fin whales and right whales [7, 16, 19, 20]. North Atlantic right whales and whale sharks both exhibit a foraging threshold associated with the density of zooplankton in the water, with feeding rarely observed when there were less than 1000 individuals m^-3^ [7, 20]. Basking sharks in the English Channel exhibit a relatively higher *in-situ* density threshold than reef manta rays, with densities of 620 mg m^-3^ of zooplankton eliciting feeding responses [16]. However, basking and whale sharks are generally much larger bodied than the reef manta rays observed in this study. Estimating an animal’s theoretical density threshold, based on energy requirements, is an alternative approach to *in-situ* sampling. In the current study, the estimated theoretical value, 25.24 mg m^-3^, was proportionately higher than that observed in the field. This contrasts with the relatively close *in-situ* and theoretical values (620 mg m^-3^ and 640 mg m^-3^, respectively) estimated for foraging basking sharks [16]. This difference may be attributable to using wet weight vs dry weight measurements, or the use of different mesh sizes for sampling. However, this might also be a consequence of inadequate estimates in the current model, suggesting a need to further investigate the energetic requirements of reef manta rays to help validate these findings. The scientific literature lacks an accurate estimation of the metabolic rate for planktivorous ray species, thus this component of our calculation relies on an estimate from other elasmobranch species [30]. However, future research could improve this estimate with the use of acceleration data loggers [41] in the field to monitor the difference in energetic output between feeding and non-feeding activity to gain insight as to whether feeding at this location is likely to be energetically profitable and/or sustainable.

If the metabolic requirements of manta rays are indeed higher than the *in-situ* findings of the current study, this could suggest that they exploit an alternative energy source to surface foraging [17]. Previous biochemical and satellite tracking data have indicated that manta rays may exploit offshore productive waters and forage on deeper zooplankton [17, 42]. LEI is located in close proximity to the continental shelf and the productive mesoscale feature of the cyclonic Capricorn Eddy [43]. Satellite tracking of manta rays from LEI revealed long periods spent offshore in this region, deep diving and movements indicative of foraging behaviour [42]. These findings, coupled with the discrepancy between foraging thresholds calculated in the current study, suggest that these large planktivores may derive a substantial part of their diet from deeper-dwelling zooplankton, and that surface feeding is only one aspect of their feeding ecology. For elasmobranchs, it is likely that diving to depth, where temperatures are below their preferred thermal range (<20°C) [4], would be energetically costly and would require a high energetic return. Thus surface feeding, such as that observed at LEI, may provide an energetic trade-off to foraging at depth.

The size of individuals in the zooplankton community did not impact whether reef manta rays were observed feeding or not, and no size threshold was required to trigger reef manta rays feeding at LEI. This finding suggests reef manta rays respond to the accumulation of zooplankton at this site, where there is an amplified abundance of organisms of all sizes, rather than a shift in community composition. This contrasts with studies on whale sharks and basking sharks that found increases of large-bodied prey items during feeding behaviour [21, 22, 44]. Quantifying the feeding behaviour of reef manta rays was beyond the scope of the current study, however we suggest future research could use drone technology to better monitor the number of manta rays in a feeding aggregation, the direction of their movements, the length of time that they feed for, and the spatial scale over which feeding takes place.

Zooplankton biomass at LEI changes rapidly over a tidal cycle, with higher biomass during the ebb to low tide. Long-term sighting records confirmed that reef manta rays are more likely to be feeding than engaged in other behaviours during these tidal phases. As the zooplankton community does not change through the tidal cycle or when reef manta rays are feeding, the associated increase in zooplankton is likely to be a result of local concentration and retention processes around the island. The circulation patterns around Whitsunday Island in the GBR lead to an accumulation of zooplankton during the flood tide [11]. At this location, a combination of the local bathymetry and tidal currents result in an area of slack water in which the zooplankton is concentrated up to 40 times (mean of 10 times) that of surrounding waters. The accumulation of zooplankton at LEI appears to happen via similar processes to that at Whitsunday Island, however further investigation of the fine-scale current scenarios and bathymetry around the island are required to elucidate this mechanism. Tidal changes have been indicated to entrain zooplankton and in turn influence manta ray feeding activity at other aggregation sites [6, 45]. We hypothesize that zooplankton is accumulating at the southern end of the island during the high tide, potentially behaving as “active drifters” rather than “passive particles” [10]. The zooplankton is then swept around the south-west during the ebbing tide, which is when we see the zooplankton biomass and the reef manta rays feeding activity increase in this area. Drogues would aid in investigating this hypothesis in the future.

Although this study was able to quantify the food environment for manta rays at LEI, the mechanism for the rapid temporal increase in zooplankton density remains unclear. The Island Mass Effect, first described by Doty and Oguri [46], attempts to explain the phenomenon of increased productivity around generally larger island masses, and has been well documented [47–49]. However, the theory has expanded to include several mechanisms that could lead to increases in primary and secondary productivity. In the case of LEI, a small island (0.45km^2^), it is the fine-scale, tidally-driven water movements that appear to be the major factor involved in entraining zooplankton and generating conditions favourable for reef manta ray feeding. An analysis of the mechanism for zooplankton accumulation at other mega-planktivore aggregation sites would be useful to help elucidate whether there are comparable environmental variables that these large animals respond to.

Whilst we have established that reef manta rays are feeding at LEI, we also observe the animals regularly engaged in courtship and cleaning behaviour at this location. Thus whether a single driver is responsible for this aggregation, or whether there is a combination of courtship, cleaning and feeding drivers, remains unclear. There has been little published information on the biomass and composition of zooplankton from manta ray aggregation sites. A future comparison of zooplankton at these different aggregation sites would help assess the importance of these sites in meeting the animals’ energetic requirements.

## Conclusion

Ephemeral resource patches, resulting from a combination of environmental factors, can have broad biological impacts on higher trophic level animals in both terrestrial and marine systems. Exploitation of transient resources is observed in baboons in South Africa feeding on seasonal flowering plants [50]; in crepuscular northern bats in Sweden feeding around streetlights during spring and autumn [51]; and seasonally in manta rays and other mega-planktivores on dense concentrations of zooplankton. The availability of such resources can affect the timing of reproductive events, migrations between habitats and animal survivorship. Thus understanding the drivers of this productivity is important, especially in aiding conservation efforts in threatened species.

Seasonal aggregation sites provide critical habitats for planktivorous elasmobranch species [52, 53] and teasing apart which habitats fulfil certain aspects of a species life history remains a challenge that research, such as the current study, is striving to address. Manta rays and other large-bodied planktivores aggregate in small areas in comparison to their home range, and sit at the apex of a relatively short food chain. Aggregations of planktivores may be a good indicator of health at the base of these food chains in these locations [54]. Given the predicted changes to the distribution of the plankton community in response to climate change [9], monitoring the movements and migrations of aggregations of planktivorous elasmobranchs over time may be important for understanding productivity changes within these systems.

Manta ray species are listed as vulnerable to extinction on the IUCN Red List of Threatened Species [52]. Over the past decade, there has been considerable research effort to understand the basic ecology and biology of these animals to inform their conservation. A remaining gap, however, is an understanding of their movement ecology in relation to foraging. Conserving highly mobile species can be particularly challenging [52], however, analysis of species critical habitats and drivers for their migrations can help inform conservation planning [53]. This study has described the feeding ecology of reef manta rays in relation to prey density requirements and local environmental variables at a key aggregation site. This knowledge can be applied to help predict critical habitats for this species in relation to their foraging preferences and oceanographic parameters. Such information can aid decision making for the long-term protection of this highly mobile species [55]. Identifying critical habitats provides information for focusing future research such as population estimates and genetics studies, vital for improving the conservation of vulnerable species [52].

## Supporting Information

S1 File Underlying dataset for 2014 reef manta ray feeding study at Lady Elliot Island. (XLSX)
